# Supplementary material for: Predicting visual function by interpreting a neuronal wiring diagram
Source: Nature. 2024 Oct 2;634(8032):113–23. doi: 10.1038/s41586-024-07953-5 (PMC11446822; doi:10.1038/s41586-024-07953-5)
Supplement: Supplementary file 6 — CRF and ERF predictions for individual TmY4 and TmY9 cells. Analogous to Supplementary Data 3, but for TmY target types. Shown are the top four monosynaptic pathways, the strongest pathway passing through each of the top ten intermediary types (ranking from Extended Data Fig. 7), and the trisynaptic pathway Tm1–TmY–Dm3–TmY (see the section entitled Prediction of spatial normalization). [file 41586_2024_7953_MOESM6_ESM.zip › DataS4/TmY9q/720575940641610203.pdf]

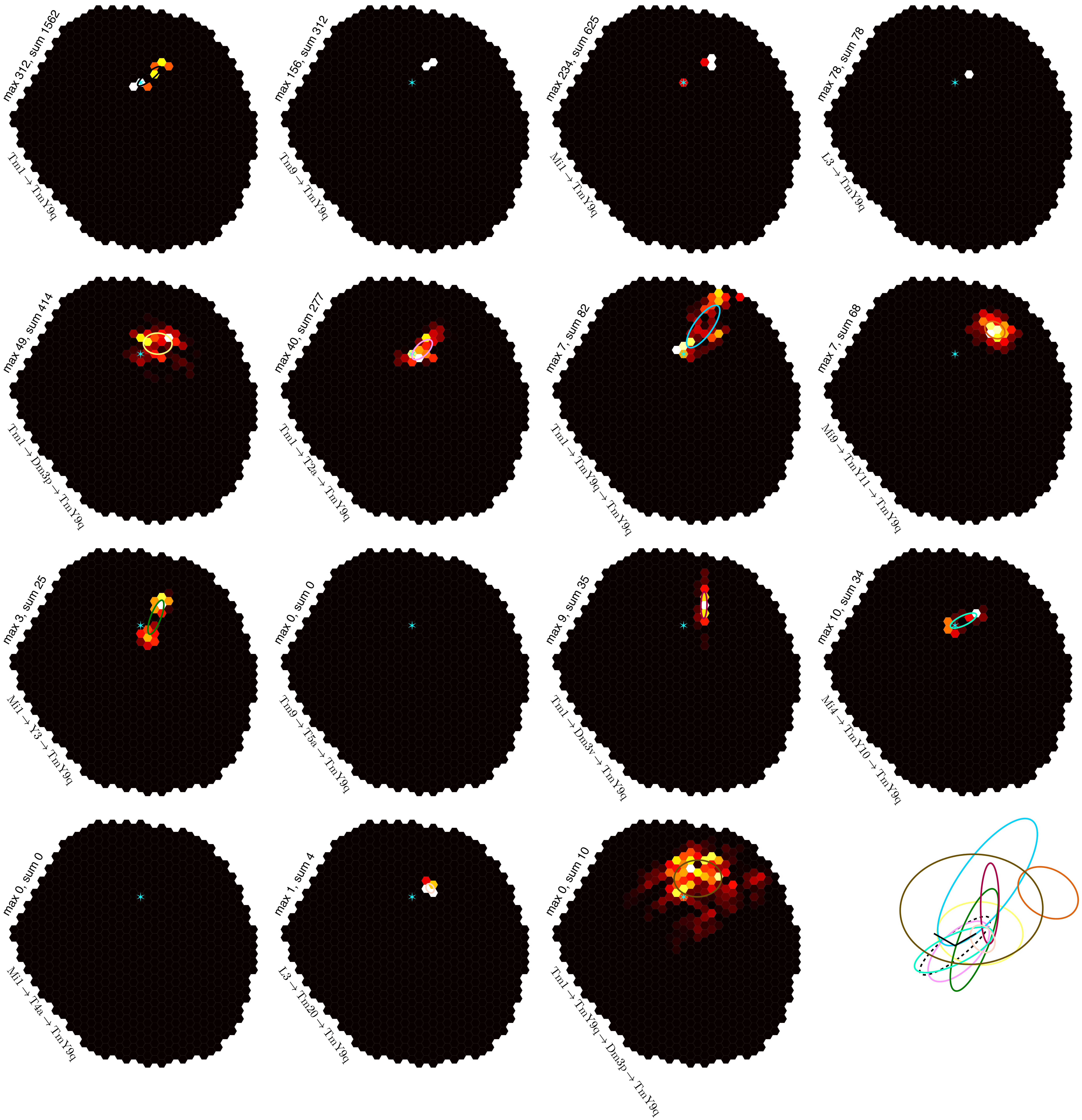

max 312, sum 1562
Tm1 → TmY9q
max 156, sum 312
Tm9 → TmY9q
max 234, sum 625
Mi1 → TmY9q
max 78, sum 78
L3 → TmY9q
max 49, sum 414
Tm1 → Dm3p → TmY9q
max 40, sum 277
Tm1 → T2a → TmY9q
max 7, sum 82
Tm1 → TmY9q → TmY9q
max 7, sum 68
Mi9 → TmY11 → TmY9q
max 3, sum 25
Mi1 → Y3 → TmY9q
max 0, sum 0
Tm9 → T5a → TmY9q
max 9, sum 35
Tm1 → Dm3v → TmY9q
max 10, sum 34
Mi4 → TmY10 → TmY9q
max 0, sum 0
Mi1 → T4a → TmY9q
max 1, sum 4
L3 → Tm20 → TmY9q
max 0, sum 10
Tm1 → TmY9q → Dm3p → TmY9q
